# New perspectives from South-Y-East, not all about death. A report of the 12^th^ lnternational Meeting on Yeast Apoptosis in Bari, Italy, May 14th-18th, 2017

**DOI:** 10.15698/mic2018.02.616

**Published:** 2018-01-16

**Authors:** Nicoletta Guaragnella, Mariarita Stirpe, William Burhans, Manuela Côrte-Real, Campbell Gourlay, Paula Ludovico, Frank Madeo, Dina Petranovic, Joris Winderickx, Cristina Mazzoni, Sergio Giannattasio

**Affiliations:** 1 Institute of Biomembranes, Bioenergetics and Molecular Biotechnologies, CNR, Bari, Italy.; 2 Pasteur Institute-Cenci Bolognetti Foundation, Department of Biology and Biotechnologies "C. Darwin", Sapienza University of Rome, Rome, Italy.; 3 Department of Molecular and Cellular Biology, Roswell Park Cancer Institute, Buffalo, NY, USA.; 4 Centre of Molecular and Environmental Biology (CBMA), Department of Biology, University of Minho, Braga, Portugal.; 5 Kent Fungal Group, School of Biosciences, University of Kent, Canterbury, UK.; 6 Life and Health Sciences Research Institute (ICVS), School of Health Sciences, University of Minho, Braga, Portugal.; 7 ICVS/3B’s - PT Government Associate Laboratory, Braga/Guimarães, Portugal.; 8 Institute of Molecular Biosciences, University of Graz, Graz, Austria.; 9 BioTechMed Graz, Graz, Austria.; 10 Systems and Synthetic Biology, Department for Biology and Biological Engineering, Chalmers University of Technology, Gothenburg, Sweden.; 11 Functional Biology, KU Leuven, Leuven, Belgium.

**Keywords:** yeast, cell death, aging, autophagy, mitochondria, stress response, system biology, drug discovery, disease model, bioprocess engineering

Over the last 14 years, the field of yeast regulated cell death (RCD) has been expanding to more and more biomedical research themes, including aging, human diseases, cell stress response, metabolism and systems biology. The 12^th^ International Meeting on Yeast Apoptosis (IMYA12), which was held in Bari, Italy from May 14^th^ to 18^th^, 2017, nicely reflected this trend. This year, more than 100 participants, among which senior and young scientists from Europe, USA, North Africa and Japan, had an intense and open exchange of achievements and ideas. This open and informal communication among researchers has been a constant hallmark of all the IMYA meetings. The global yeast death community was embraced and inspired by the lively and warm atmosphere of Bari, the capital city of Apulia, and its beautiful surroundings, with colorful landscapes, historical and artistic heritage, tastes and scents that reflect the interface between Eastern and Western culture.

The meeting started with the keynote lecture of **Frank Madeo** (Austria), a pioneer of yeast apoptosis studies. He presented data on the anti-aging effects of spermidine in different model organisms, including yeast, nematodes and flies. He demonstrated that this ubiquitous polycation induces autophagy and significantly reduces age-related oxidative protein damage in mice. Spermidine supplementation was also shown to have a strong cardio protective effect on diastolic parameters. These findings support the evidence that autophagy mediates cytoprotection against a variety of noxious agents, thereby conferring longevity when induced at the whole-organism level. Outstanding scholars gave their plenary lectures in several thematic sessions as follows.

## FUNGAL CELL DEATH PATHWAYS

The perception of mammalian "cell death" has evolved from being a passive incident to an active process as supported by the characterization of several active death pathways, such as apoptosis, necroptosis, or pyroptosis. In contrast, although the evidence of apoptosis in dying fungal cells is clear, less is known about its molecular mechanisms and the impact of the environmental cues on cell fate control. **Marie Hardwick** (USA), the first speaker of the opening session on fungal cell death pathways reported the identification of certain death-resistant deletion strains through a genome wide screen analysis in *Saccharomyces cerevisiae*. These strains were knockouts for all subunits of a vesicle trafficking complex involved in responding to amino acid levels, thus supporting the evidence that yeast cell death and nutrient-sensing pathways merge and can affect survival following stress conditions.

**Campbell Gourlay** (UK) presented results on the conserved link between environmental signals, cytoskeletal integrity and cell fate control. Particularly, he showed that defects in actin regulation lead to the inability to control the fidelity of MAPK signaling pathways. He observed that the yeast filamentous growth and mating MAPK pathways become inappropriately activated in response to a reduction in actin dynamics and that actin stabilization leads to the constitutive activation of the MAPK controlled cell wall integrity pathway. He also observed that the de-regulation of MAPK signaling leads to vacuole dysfunction, ROS production and cell death that can be reversed by porin deletion.

**Katrina Cooper** (USA), who is working on cyclin C-mediated cell death, proposed a model in two steps for cyclin C release from the nucleus to the cytoplasm, representing a key cell-fate checkpoint control. She found that first cyclin C is phosphorylated by the MAP kinase Slt2 permitting its disassociation from the Mediator component Med13 and after that Med13 destruction allows full cyclin C release and prevents re-accumulation of the cyclin in the nucleus.

**Manuela Côrte-Real** (Portugal) analyzed the selective anticancer activity of lactoferrin showing that this glycoprotein activates a mitochondria- and caspase-dependent apoptotic process in yeast, which led to the hypothesis that lactoferrin could act as a V-H^+^-ATPase inhibitor in highly metastatic cancer line cells. In addition, she showed that bovine lactoferrin causes intracellular acidification in metastatic breast cancer cells. Her data demonstrate how yeast can provide relevant clues in the identification of the molecular target/mechanisms of action of lactoferrin.

## MITOCHONDRIAL FUNCTION AND PATHOPHYSIOLOGY

During cell division, the partitioning of cell organelles involves both stochastic and ordered mechanisms and budding yeast is a useful model to study organelle inheritance during asymmetric cell division. **Benedikt Westermann** (Germany) added information to this understanding by presenting data on the unexpected link between mitochondrial transport and fusion. Genetic analysis, live cell microscopy and simulations *in silico* showed that this link is critical for a correct mitochondrial partitioning and that mitochondrial quantity causes a severe decline of replicative lifespan of daughter cells. This suggests that coordination of mitochondrial transport, fusion, and fission is critical for asymmetric division and rejuvenation of daughter cells.

**Michal Eisenberg-Bord** (Israel) discussed lipid droplets and their key functions in bioenergetics homeostasis and membrane biosynthesis. In particular, she focused on a specialized lipid droplet subpopulation equipped with a unique set of surface proteins that is localized in proximity of the nucleus vacuole junction. By using high-throughput screening, she identified two Lipid Droplet Organization (Ldo) factors, which are required for proper localization of Pdr16 residing in this lipid droplet subpopulation.

## METABOLIC REGULATION OF CELL STRESS RESPONSE

As metabolism represents the core of cellular functions and since most cellular processes interact with metabolism, a highly complex network is established. **Jens Nielsen** (Sweden) addressed how different parts of cellular metabolism are connected, how metabolism can be modelled at the genome-scale and how incorporation of protein crowding and proteome homeostasis may be key determinants for cellular function. He also showed how regulation of metabolism can be studied using different omics analysis.

**Hiroshi Takagi** (Japan) presented data on the signaling molecule nitric oxide (NO) and the flavoprotein Tah18, which is involved in nitric oxide synthase-like activity. He reported that cell death induced by Tah18-dependent NOS activity can be prevented by enhancing the interaction between Tah18 and its molecular partner Dre2. Appropriate nitric oxide levels were found to confer cellular tolerance either to high temperature, via Mac1-mediated Sod1 activation, or to low levels of hydrogen peroxide. In contrast, under severe stress conditions, such as high levels of hydrogen peroxide, excess NO may induce cell death. Tah18-dependent NO production may thus exhibit two opposite effects in yeast, similar as in mammalian cells.

## AUTOPHAGY/MITOPHAGY

Autophagy is a conserved process that functions during cellular stress and nutrient starvation as part of an adaptive response to maintain homeostasis and quality control. **Francesco Cecconi** (Italy) discussed the role of AMBRA1, the activating molecule in beclin 1-regulated autophagy, and he placed this in the context of evolution and development. Beyond AMBRA1’s recognized role in embryonic development, neurological disorders and carcinogenesis, he showed that AMBRA1 can coordinate a cell response to starvation or other stresses by translocation of the autophagosome core complex to ER, by regulative ubiquitination and stabilization of the kinase ULK1, and by selective mitochondria removal and cell cycle down-regulation. AMBRA1 appears also to be targeted by different regulators, such as Culling-dependent degradation, caspase cleavage and several modifications, from phosphorylation to ubiquitination.

**Ted Powers** (USA) discussed the role of the rapamycin-insensitive TORC2 as a positive regulator of autophagy, specifically under amino acid starvation. He described the architecture and the activity of this regulatory pathway, showing that TORC2, through its downstream target kinase Ypk1, inhibits calcineurin. On the other hand, calcineurin activation requires Mid1, a calcium channel regulatory protein and when TORC2-Ypk1 signaling is compromised, the normal mitochondrial respiration is perturbed resulting in the accumulation of reactive oxygen species and the inhibition of the general amino acid control and autophagy.

## CELLULAR AGING

Similar as multicellular organisms, growing yeast cells are able to differentiate in organized communities, such as colonies. In such colonies, cells initiate various metabolic reprogramming leading to the formation of cell subpopulations that mutually interact in order to fulfill specific tasks. **Zdena Palková** (Czech Republic) focused on the regulatory role of mitochondria and certain mitochondrial pathways during cell subpopulation longevity as well as on the regulation of metabolic differentiation and the production of specific metabolic proteins and transporters in specific cell subpopulations.

It is well known that yeast offers an excellent tool to study the molecular basis of human diseases as it: allows mimicking a variety of physio-pathological aspects under different environmental conditions. By avoiding complex ethical issues associated with mammalian models or human subjects, the use of yeast models enable the discovery of novel targets via high-throughput screens, thereby accelerating therapeutic research provided that such targets are further validated in more complex models. **Tiago Outeiro** opened this part of IMYA12 with an inspiring talk in memory of his mentor Susan Lindquist, who pioneered on the use of budding yeast as model system for studying human disease, evolution, and biomaterials. The following sessions then ensued.

## PROTEIN AGGREGATION AND TOXICITY

Studies in* S. cerevisiae* have significantly contributed to our understanding of the cellular mechanisms underlying neurodegenerative diseases, such as Parkinson’s (PD) and Alzheimer’s diseases (AD), for which the pathobiology remains largely unclear. **Tiago Outeiro** (Germany) has investigated the role of alpha-synuclein in PD in a yeast model. He presented data about the effect of post-translational modifications, such as phosphorylation and glycation, on alpha-synuclein aggregation and toxicity. His findings on phosphorylation of alpha-synuclein at a specific serine residue have been validated in mammalian cell models and may open novel perspectives for therapeutic intervention in synucleopathies.

**Dina Petranovic** (Sweden) presented results on the expression in yeast of two forms of the amyloid β-peptide (Aβ), which typically deposits in the amyloid plaques found in AD brain, but whose mechanisms of toxicity are not fully understood. By comparative analysis of the physiology, molecular markers and genome-wide transcriptional responses in strains constitutively expressing either Aβ1-42 or Aβ1-40, she identified some mechanisms that seem to be implicated in the toxicity of Aβ peptides. She also described recent findings on a mutant protein, named ubiquitin B protein+1 (UBB+1), which accumulates in an age-dependent manner and appears to correlate with AD. Differently from other data, low level of UBB+1 can induce a protective response assisting yeast cells to cope better with misfolded proteins during chronological aging, including the reduction of the Aβ toxicity associated to lower levels of Aβ aggregates.

## YEAST MODELS OF HUMAN DISEASES

**Joris Winderickx** (Belgium) has been using yeast as a model to decipher fundamentals of age-associated human disorders. Particularly, he focused on the interplay of PKA-, TORC1- and Sch9- controlled nutrient-sensitive pathways that have a profound impact on growth, stress resistance and longevity. He gave an overview on recent findings about the connections of intracellular pH and calcium homeostasis with these pathways and he discussed their effect on the overall cell physiology.

**Richard Y. Zhao** (USA) presented evidence that fission yeast can be used as a surrogate system for the rapid identification and genome-wide functional analysis of Zika virus (ZIKV) proteins. ZIKV causes various congenital neurologic disorders including microcephaly in newborns and Guillain-Barré Syndrome in adults. Through the yeast studies, seven ZIKV proteins were identified that cause different levels of cytopathic effects including cell growth restriction, cell cycle dysregulation and cell death. Those initial findings generated from yeast provide a primary reference for future mammalian studies on their potential links to ZIKV-induced neurologic disorders. Ongoing mammalian translation studies were also presented and discussed.

**Ali Gargouri** (Tunisia) described the effects of p53 ectopic expression in *S. cerevisiae* by illustrating different aspects: the occurrence and analysis of apoptosis, the structural and functional connection with mitochondria, the impact on gene expression and screening of intragenic or over-expressing *p53*-inactivating mutations. He also showed the selection of biomolecules suppressing the p53-mediated cell death, including a very potent macromolecule, extracted from *Nigella sativa* that is able to rescue apoptosis without affecting p53 expression.

**Carlo Bruschi** (Austria) underlined the complex apoptotic dynamics seen following chromosome translocation events that depend on the subsequent genomic rearrangements and epigenetic alteration of the primary homeostatic structure of the chromatin. In particular, he reported death rates and expression profiles of apoptosis-related genes in yeast cells in which the primary event of chromosome translocation was induced *ad hoc* by Bridge-Induced Translocation (BIT) technology. He also showed that the BIT system was successfully applied to reproduce in yeast precisely the peculiar chromosome translocation associated with acute myeloid leukemia and that this yeast model can be used to test the constitutive expression of human *P53*.

## YEAST-BASED DRUG DISCOVERY

*S. cerevisiae *has been successfully used as a model organism in phenotypic screens aiming to identifying molecules of pharmacological interest or with anti-aging properties, to understand mechanism of action of drug or to reveal novel drug targets and target pathways. **Ralf Braun** (Germany) presented results on the expression and clearance of the nuclear RNA-binding neurotoxic protein, TDP-43, which accumulates and aggregates in the cytoplasm of patients affected by motor neuron disorder amyotrophic lateral sclerosis. He demonstrated that TDP-43 interferes with its own degradation via the lysosomal clearance pathway, potentially by entrapping its pivotal components. Modulating the fine balance between cell survival and protein degradation systems might delay the loss of neuronal activities during disease progression.

**Paula Ludovico** (Portugal) showed how metabolite supplementation, such as certain amino acids, can have anti-aging effects when cells suffer proteotoxic stress elicited by the heterologous expression of human α-synuclein (α-syn). These effects are associated with a decreased accumulation of reactive oxygen species, Sch9-mediated increased superoxide dismutase activity and alterations in the mitochondrial network. These findings might be an attractive mechanism to rescue cells from α-syn mediated toxicity and for future pharmacological research designed to improve the prognosis of age-associated diseases.

## SYSTEMS-BASED MODEL OF CELL DEATH AND AGING PROCESS

Systems biology studies in yeast are supporting the development and understanding of the complexity and interplay of biological networks with their dynamics, basic principles, rules and balanced orchestrated functions in direct interaction with the environment.** Charles Boone** (Canada) presented the quantitative genetic interaction data produced by genome-scale Synthetic Genetic Array (SGA) experiments with *S. cerevisiae. *He outlined the functional interactions and the relationships genotype-phenotype, revealing the potential of genetic interactions for assembly of a hierarchical model of cell function, including modules corresponding to protein complexes and pathways, biological processes, and cellular compartments. He also introduced the *in fieri* work on the extent and composition of the global yeast trigenic interaction network.

**Duccio Cavalieri** (Italy) discussed the physiological significance of PCD in the framework of ecological interactions of "natural" yeast strains with different life span and different levels of toxins production, such as ethanol and acetic acid, in sugar rich environment. He presented evidences for the role of released Hsp12p and Pau5p in modulating cell growth under the effect of quorum sensing molecules and determining yeast community level fitness in different ecological settings.

## STRESS RESPONSES FOR IMPROVEMENT OF BIOPROCESSES

Several industrial yeast-based processes, such as the production of ethanol from lignocelluloses or wine making, are inhibited by the accumulation of organic acids, including acetic acid, which is known to inhibit metabolism and affect cell viability. Thus, one of the central issues for the improvement of their robustness is to gain insight into the molecular mechanisms of acetic acid toxicity.** Maurizio Bettiga** (Sweden) presented results on the relationships between plasma membrane properties and acetic acid stress tolerance. He demonstrated that an increase of the sphingolipids content in the plasma membrane is associated with acetic acid stress response in *Zygosaccharomyces bailii*. These studies point to the possibility to engineer the membrane composition of an industrial yeast strain towards reduced permeability as to obtain higher acetic acid tolerance.

## CONCLUDING REMARKS

With 37 selected oral presentations and 26 posters, IMYA12 has been a showcase for the latest advances in the knowledge on the molecular basis of many fundamental cellular processes governing cell homeostasis. IMYA12 also presented cutting-edge achievements in the exploitation of yeast as outstanding model for deciphering at the system level different aspects related to biomedical and bio-industrial science. With the aim of witnessing this new fascinating era of yeast research, the IMYA Scientific Advisory Board has decided to update the title of the conference series into *International Meeting on Yeast Aging and Apoptosis,* though maintaining the winning-horse acronym IMYA. The next meeting, i.e. IMYA13, will be organized in Leuven, Belgium. Thus, "*tot binnenkort in België"*!

